# High-precision monitoring of and feedback control over drug concentrations in the brains of freely moving rats

**DOI:** 10.1126/sciadv.adg3254

**Published:** 2023-05-17

**Authors:** Julian Gerson, Murat Kaan Erdal, Matthew H. McDonough, Kyle L. Ploense, Philippe Dauphin-Ducharme, Kevin M. Honeywell, Kaylyn K. Leung, Netzahualcoyotl Arroyo-Curras, Jenny M. Gibson, Nicole A. Emmons, Wendy Meiring, Joao P. Hespanha, Kevin W. Plaxco, Tod E. Kippin

**Affiliations:** ^1^ Department of Psychological and Brain Sciences, University of California, Santa Barbara, CA 93106, USA.; ^2^ Neuroscience Research Institute, University of California, Santa Barbara, CA 93106, USA.; ^3^ Institute for Collaborative Biotechnologies, University of California, Santa Barbara, CA 93106, USA.; ^4^ Department of Electrical and Computer Engineering, University of California, Santa Barbara, CA 93106, USA.; ^5^ Department of Statistics and Applied Probability, University of California, Santa Barbara, CA 93106, USA.; ^6^ Department of Chemistry and Biochemistry, University of California, Santa Barbara, CA 93106, USA.; ^7^ Département de chimie, Université de Sherbrooke, Sherbrooke, QC J1K 2R1, Canada.; ^8^ Department of Pharmacology and Molecular Sciences, Johns Hopkins University School of Medicine, Baltimore, MD 21205, USA.; ^9^ Department of Molecular Cellular and Developmental Biology, University of California, Santa Barbara, CA 93106, USA.

## Abstract

Knowledge of drug concentrations in the brains of behaving subjects remains constrained on a number of dimensions, including poor temporal resolution and lack of real-time data. Here, however, we demonstrate the ability of electrochemical aptamer-based sensors to support seconds-resolved, real-time measurements of drug concentrations in the brains of freely moving rats. Specifically, using such sensors, we achieve <4 μM limits of detection and 10-s resolution in the measurement of procaine in the brains of freely moving rats, permitting the determination of the pharmacokinetics and concentration-behavior relations of the drug with high precision for individual subjects. In parallel, we have used closed-loop feedback-controlled drug delivery to hold intracranial procaine levels constant (±10%) for >1.5 hours. These results demonstrate the utility of such sensors in (i) the determination of the site-specific, seconds-resolved neuropharmacokinetics, (ii) enabling the study of individual subject neuropharmacokinetics and concentration-response relations, and (iii) performing high-precision control over intracranial drug levels.

## Teaser

A sensing platform permits real-time, high precision monitoring and control of in-brain drug concentration of behaving subjects.

## INTRODUCTION

Given that the impact of a drug on brain function depends critically on its in-brain pharmacokinetics, accurate, and highly time-resolved measurement of in-brain drug concentrations will be necessary to achieve a detailed understanding of drug-brain interactions. For example, the greater abuse potential of injecting rather than oral ingestion or “snorting” of psychotropic drugs is thought to occur because in-brain drug levels peak a few tens of seconds faster in the former (*1*–*3*). The induction of anesthesia likewise typically requires just seconds (e.g., the time required to count backward from 100 to 90). Both observations highlight the important role that seconds-resolved in-brain drug measurements will play in advancing our understanding of psychopharmacodynamics.

In contrast to the few tens of seconds time course of many psychoactive substances, even the best-resolved prior studies of their pharmacokinetics are typically (*1*) only minutes resolved and (*2*) not real time (*4*, *5*), with the latter precluding their application to closed-loop feedback control over drug levels. These issues arise because current methods of measuring drugs in the brain are largely limited to postmortem sample collection, positron emission tomography (PET), or microdialysis (*6*, *7*), all of which fail to achieve the performance necessary to address many key questions in behavioral neuropharmacology. Postmortem measurements, for example, provide only a single time point and typically lack spatial resolution (specimens are often homogenized). PET, in contrast, supports the real-time measurement of drug time courses but is limited to those molecules for which high-speed isotopic labeling protocols are available, requires constrained subjects, and suffers from poor (typically minutes) resolution (*8*–*10*). Microdialysis, which also supports the collection of multiple, time-resolved measurements, not only provides exceptional versatility in molecular detection and supports studies of freely moving subjects (*11*, *12*) but also suffers from generally poor temporal resolution. Specifically, while a single group (*13*) has used microdialysis to achieve 2-s resolution measurements of in-brain amino acid concentrations (a feat that does not appear to have since been repeated) and a small number of others have reached tens of seconds resolution [e.g., (*14*, *15*), see (*16*) for recent review], such studies remain far from commonplace, much less “turn key.” For example, a recent survey of a representative set of 64 in-brain microdialysis studies found the best resolved of these used 5-min sampling and most (82%) used sampling times of 20 min or greater (*17*). In addition, although numerous authors have described “on-line” setups in which dialysate is delivered directly to analytical instrumentation for measurement (*18*), minute-long sample transport and analysis times mean that even these microdialysis studies are not meaningfully real-time, precluding their application to emerging engineering approaches, such as feedback control of in vivo drug concentrations (*19*, *20*) and closed-loop optogenetics (*21*, *22*).

**Fig. 1.**
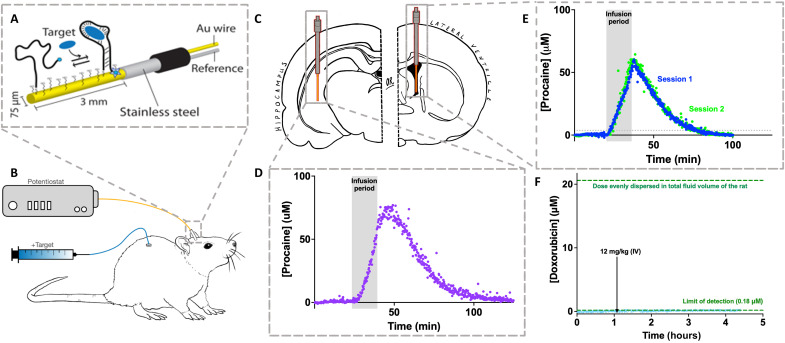
Adaptation of EABs to in-brain measurements. (**A**) EABs are composed of a redox reporter–modified, target-binding aptamer covalently attached to a gold electrode. Binding-induced folding of the aptamer leads to an easily measured change in electron transfer that is quantitatively and monotonically related to target concentration (fig. S1). For the in-brain studies reported here, we fabricated EAB sensors on a 75-μm-diameter, 3-mm-long gold electrode extending from the end of a 22-gauge stainless steel cannula that acts as both a guide and a counter/pseudo-reference electrode. (**B** and **C**) This is inserted into the brain region of interest using a stereotaxically implanted 19-gauge guide cannula. We use a chronic, indwelling jugular catheter for drug delivery. (**D**) Using these intracranial EAB sensors, we can measure procaine levels in specific brain regions with resolution of order 10 s. Shown here, for example, are measurements performed in the hippocampus before, during, and after a total dose of 80 mg/kg infused at 5 mg/kg/min. (**E**) To demonstrate the reproducibility of intracranial EAB sensors, here, we have performed measurements in the lateral ventricle of a single rat on two occasions 6 days apart, each time challenging the animal with a total dose of 80 mg/kg infused at 5 mg/kg/min. These challenges lead to effectively indistinguishable maximum concentrations (*C*_max_ = 63.4 ± 0.3 and 65.7 ± 0.3 μM for sessions 1 and 2, respectively) and closely similar postinfusion pharmacokinetics (*t*_1/2_ = 12.1 ± 0.1 and 10.9 ± 0.1 min, respectively). (**F**) A control experiment using a sensor against the chemotherapeutic doxorubicin, which, unlike procaine, does not cross the blood-brain barrier (*36*, *37*), confirms that the placement of these sensors leaves the barrier intact. Specifically, some 1.1 hours into this experiment, the drug was dosed intravenously (IV) at 12 mg/kg, a dose that would have saturated the sensor were the drug able to cross into the brain (see fig. S2).

In contrast to in-brain measurements of drugs, current methods of measuring some neurotransmitters in the brain achieve far superior temporal resolution. Specifically, the direct electrochemical detection of monoamines and the enzymatic electrochemical detection of glutamate and acetylcholine commonly achieve resolution of ~1 s (*23*). [Note that, while the interrogation frequency of such sensors is often >>1 Hz, this is not synonymous with their time resolution, which is limited to a few seconds by the slow diffusion of target to the sensor surface; see, e.g., figure 1 of (*24*), table 1 of (*25*), and table 2 of (*16*)]. These approaches, however, depend on the electrochemical or enzymatic reactivity of their targets and thus cannot be generally adapted to the measurement of most psychoactive drugs (the one exception being alcohol). Here, however, we describe the adaptation of electrochemical aptamer-based (EAB) sensors (Fig. 1), a versatile, receptor-based, electrochemical detection approach that is independent of the reactivity of its targets [e.g., (*26*, *27*)], to the problem of performing seconds-resolved, real-time, multihour measurements of and feedback control over the intracranial concentration of a psychoactive drug in freely behaving rats.

## RESULTS

EAB sensors are composed of a redox reporter–modified aptamer (nucleic acid–based receptors selected in vitro to bind specific molecular targets) that is covalently attached to the surface of a gold electrode via an alkane thiol chain (Fig. 1A). A binding-induced conformational change in this aptamer alters the rate of electron transfer from the redox reporter, changing in turn the peak current observed when the sensors are interrogated using square-wave voltammetry, with the relevant interrogations requiring 10 to 11 s to perform. As the sensor responds to its target more rapidly than this (fig. S3), this interrogation time scale defines the sensor’s time resolution.

Here, we have developed an EAB sensor for the detection of the anesthetic procaine and have used it to monitor the drug intracranially in live rats. This compound, which has local, general, and epidural anesthetic properties (*28*–*31*), had previously only been measured in the brain with ~5-min temporal resolution (*5*), a time frame that is both 30-fold poorer than the results presented here and too slow to accurately resolve the drug’s pharmacokinetics or support detailed comparison of drug concentration to drug-induced behavioral effects. To develop an EAB sensor against procaine, we first modified a previously developed aptamer (*32*) to improve its affinity and signal gain (fig. S1), with the resulting sequence achieving a dissociation constant (*K*_d_) of 960 ± 30 μM (this and all other confidence intervals reflect estimated 95% confidence levels). After modifying this aptamer with a methylene blue redox reporter and a six-carbon alkane thiol, we deposit it on a 75-μm-diameter gold wire and “backfill” with 6-mercaptohexanol to form a continuous monolayer. The resulting EAB sensor easily measures the clinically relevant, 40 to 180 μM procaine concentrations seen in human plasma during the administration of this drug (*33*). For example, when challenged in vitro with 200 μM procaine in undiluted cerebrospinal fluid (CSF), the sensor responds with a ~30% change in signaling current (fig. S1). In contrast, the sensor does not measurably respond to either of the primary metabolites of procaine (fig. S1) or to any of the neurotransmitters thought to be modulated by this drug (fig. S4).

In-brain EAB sensors can be emplaced using standard surgical techniques. For insertion, animals are briefly sedated using isofluorane, and the sensor is inserted through a 19-gauge cannula that was stereotaxically implanted and cemented to the skull at least 1 week before the experiment. Given the dimensions of the probe (Fig. 1B) and the placement of the cannula (see Materials and Methods), the working electrode portion of the sensor sits entirely in the region of interest [to ensure correct placement, we fix, slice, and image all brains after the experiment (e.g., fig. S5)]. Notably, while this procedure ensures that the probes are tightly secured to the cannula and therefore to the animal’s skull, we use free-moving animals in our studies, and thus, mechanical noise is sometimes seen when the animal strikes the probe against the side of its chamber. The resulting, distorted voltammograms create substantial outliers in our recorded time-concentration plots (see, e.g., fig. S6), which we systematically remove applying a Hampel filter (with *k* = 25); doing so removes fewer than 1 in 20 data points.

When placed in freely moving, normally behaving rats, intracranial EAB sensors support seconds-resolved drug concentration measurements. For example, when placed in the hippocampus of a free-moving rat undergoing an infusion (5 mg/kg/min) to a total dose of 80 mg/kg, we see a steady rise in in-brain procaine levels to 78.6 μM (Fig. 1D). This value is similar to the only prior measurements of intracranial procaine levels that we are aware of (*5*). These experiments, which used the removal of CSF from anesthetized dogs with a sampling interval of 3 min (or greater), observed peak concentrations of 150 μM some 12 min after an infusion (50 mg/kg), values reasonably close to those we report here for the rat. As expected, given the rapid metabolism of procaine (*28*, *33*, *34*), intracranial drug levels then rapidly decrease with a half-life (*t*_1/2_) of 13.8 min. The precision of the resulting measurements is exceptional: Using noise measurements collected during the preinfusion baseline of this specific experiment, we estimate that this sensor’s limit of detection (at the level of 3 SDs above baseline) is 2.72 μM. Notably, this limit of detection is not a fundamental limitation of the EAB approach; rather, it is defined by the affinity of the aptamer for its target, the gain of the specific sensor, the noise seen in the specific in vivo setting with this specific EAB architecture (*35*), and the conservative statistical threshold we have used here.

Intracranial EAB measurements are highly reproducible. To show this, we placed a cannula to provide access to the left lateral ventricle of a single rat in a repeatable fashion. Then, on separate occasions 6 days apart, we inserted two different EAB sensors into the lateral ventricle of this animal and recorded the concentration of procaine in the CSF before, during, and after constant intravenous infusions (5 mg/kg/min) to a total dose of 80 mg/kg (Fig. 1E). In the two measurement sets, the drug rose to effectively indistinguishable maximum concentrations (*C*_max_ = 63.4 ± 0.3 and 65.7 ± 0.3 μM, respectively) shortly after the end of the infusion before then falling with *t*_1/2_s (12.1 ± 0.1 and 10.9 ± 0.1 min, respectively) that are likewise nearly identical.

Given that our goal is to measure drug transport across the blood-brain barrier, we believe that it is important to confirm that the barrier remains intact after sensor insertion. To confirm this, we performed a control experiment using the chemotherapeutic doxorubicin, a drug that is known to not cross the barrier (*36*, *37*) and for which there exists a particularly low limit of detection EAB sensor (*26*). We placed this sensor in the lateral ventricle of a rat and dosed the animal intravenously with doxorubicin levels that, were the drug to cross into the brain, would produce ~20 μM (assuming nonselective equilibration across the total fluid volume of the rat; see fig. S2), a value 100-fold above this sensor’s limit of detection. Despite this, we did not see any measurable change in sensor output (Fig. 1F), confirming that our experiments do not significantly disrupt the barrier, an observation that is perhaps not unexpected given that EAB sensors should be no more disruptive than the well-established microdialysis approaches that have preceded them.

**Fig. 2.**
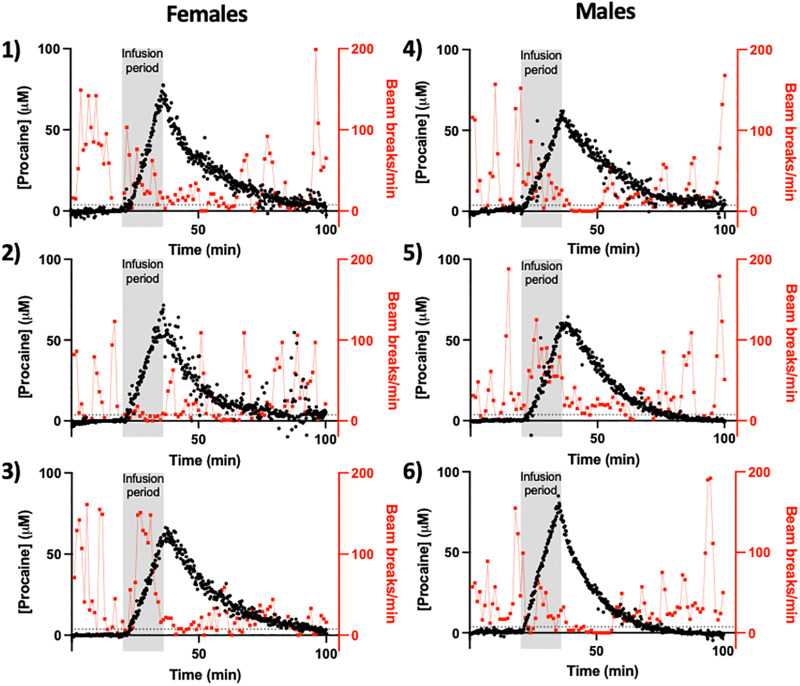
Individual subject neuropharmacokinetics and response to moderate-dose procaine. Here, we have measured procaine levels (black) in the lateral ventricles of six freely moving rats while simultaneously monitoring their behavior (in red is beam breaks per minute). In this experiment, male and female were given a continuous intravenous infusion (80 mg/kg) at a rate of 5 mg/kg/min; the resulting 16-min infusion periods are denoted by the vertical gray bars. In these measurements, we first detected the drug at 3.7 μM (denoted by horizontal dashed lines), which is 3 SDs above the baseline (i.e., at the 99.7% confidence level) for the noisiest (i.e., worst) sensor presented here, some 71 to 175 s of the initiation of the infusion. In each case, the level of the drug in the ventricle then rises monotonically for the duration of the infusion before rapidly falling as the drug is subsequently metabolized. Note: The animal number provided next to each panel is consistent between all figures and tables.

**Fig. 3.**
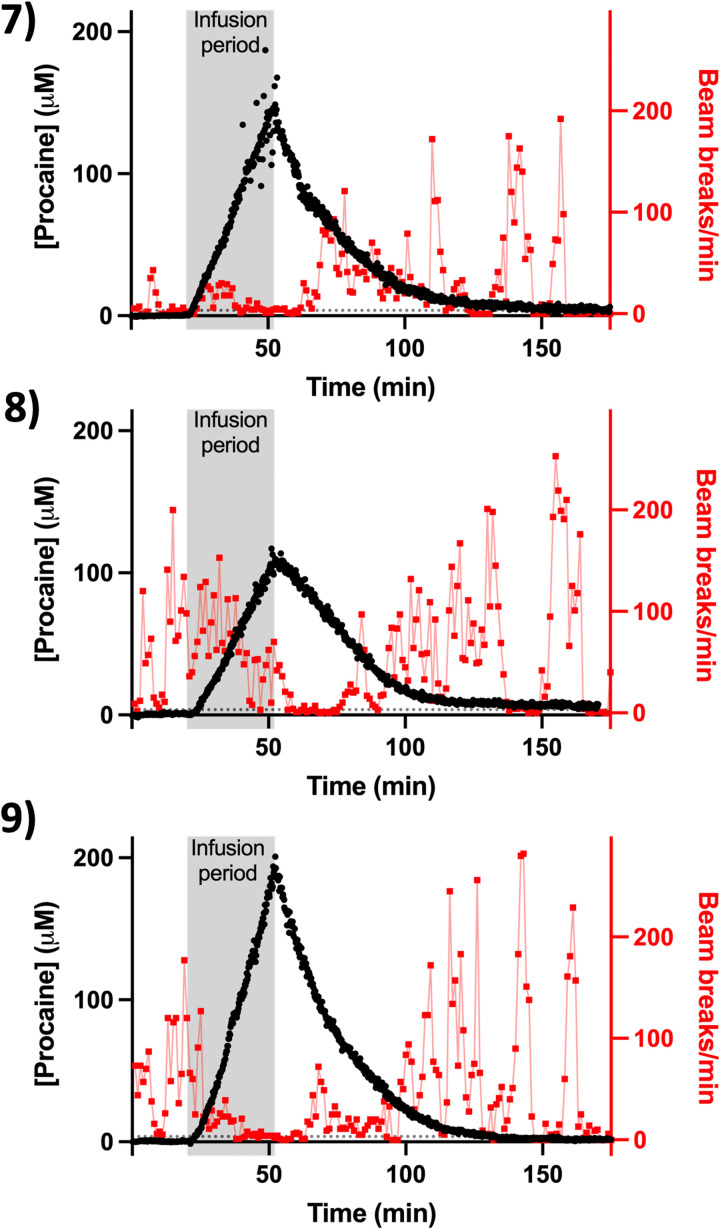
Individual subject neuropharmacokinetics and response to high-dose procaine. Here, we used EAB sensors to monitor intracranial procaine after larger (longer) infusions (32 min at 5 mg/kg/min) and, consequently, higher total doses (160 mg/kg). In these experiments, we first detected the drug at our 3.7 μM, 3-σ threshold (denoted with dashed lines), within 174 to 220 s of the initiation of the infusion.

The exceptional resolution of EAB sensors renders it possible to resolve the pharmacokinetics of individual animals with good statistical significance. To illustrate this, we placed sensors into the lateral ventricles of nine animals [we used the ventricles because the exact site of action of anesthetics, such as procaine, remains unknown (*38*)]. Using these, we then monitored the drug in the CSF before, during, and after a constant intravenous infusion (5 mg/kg/min) to total dosing of 80 or 160 mg/kg. Across these nine animals, procaine first reaches 3.7 μM (which is the 3-σ = 99.7% confidence limit of detection seen for the noisiest of the dozen sensors used in the work described here) in the ventricles some 71 to 220 s after the initiation of infusion. For six of these animals, we dosed for 16 min to a total of 80 mg/kg. In these animals, we saw the drug rise to peak concentrations of 56 to 80 μM shortly after the end of the infusion (Fig. 2). In contrast, for the three animals, we dosed to a total of 160 mg/kg, the corresponding peak concentrations range from 126 to 197 μM (Fig. 3). In all nine animals, the rapid metabolism of procaine again causes its concentration to fall shortly after the cessation of the infusion.

The unprecedented time resolution of EAB-derived drug measurements provides hundreds of concentration values during the ~100 to 150 min between the initiation of drug exposure and the elimination of the drug from the brain to undetectable levels. These unprecedentedly highly time-dense data render it possible to estimate key pharmacokinetic variables, such as the *C*_max_ and the elimination *t*_1/2_, with precision typically better than 1% (at 95% confidence intervals). With this level of precision, we can easily identify pharmacokinetic differences between individual animals with good statistical confidence. To illustrate these points, we explored the single-exponential drug kinetics seen following the cessation of infusion (Figs. 2 and 3). For the infusions (80 mg/kg), the *t*_1/2_s of these decays range from 8.6 ± 0.1 to 15.7 ± 0.1 min (Table 1), indicating small but nevertheless highly statistically significant animal-to-animal variation in procaine metabolism. Following the infusion (160 mg/kg), the observed *t*_1/2_s range from 15.9 ± 0.1 to 20.3 ± 0.1 min. Notably, the 12.6 ± 2.1 min average *t*_1/2_ seen across the six animals that received doses of 80 mg/kg is slightly, but statistically significantly, shorter than the 18.1 ± 2.5 min average seen for the three animals that received the 180 mg/kg dose, suggesting that the esterase that degrades procaine may approach saturation at higher the drug concentrations achieved in the latter experiments, slowing the drug’s elimination.

**Table 1.** Neuropharmacokinetics parameters for individual subjects.

Animal number	Time to first detection* (min)	*C*_max_ (μM)	*t*_1/2_ **(min)**
**Lateral ventricle, 80 mg/kg dosing**			
**1**	1.18	66.4 ± 0.3	13.8 ± 0.1
**2**	1.98	55.9 ± 0.3	12.0 ± 0.1
**3**	2.72	66.3 ± 0.3	14.3 ± 0.1
**4**	2.92	60.6 ± 0.3	15.7 ± 0.1
**5**	1.23	65.7 ± 0.3	10.9 ± 0.1
**6**	1.6	79.5 ± 0.3	8.6 ± 0.1
**Lateral ventricle, 160 mg/kg dosing**			
**7**	2.9	197.2 ± 0.3	15.9 ± 0.1
**8**	3.67	126.0 ± 0.2	20.3 ± 0.1
**9**	2.17	154.5 ± 0.2	18.2 ± 0.1

*Defined as the time taken to surpass the limit of detection, here defined as 3.7 μM (see the main text).

**Fig. 4.**
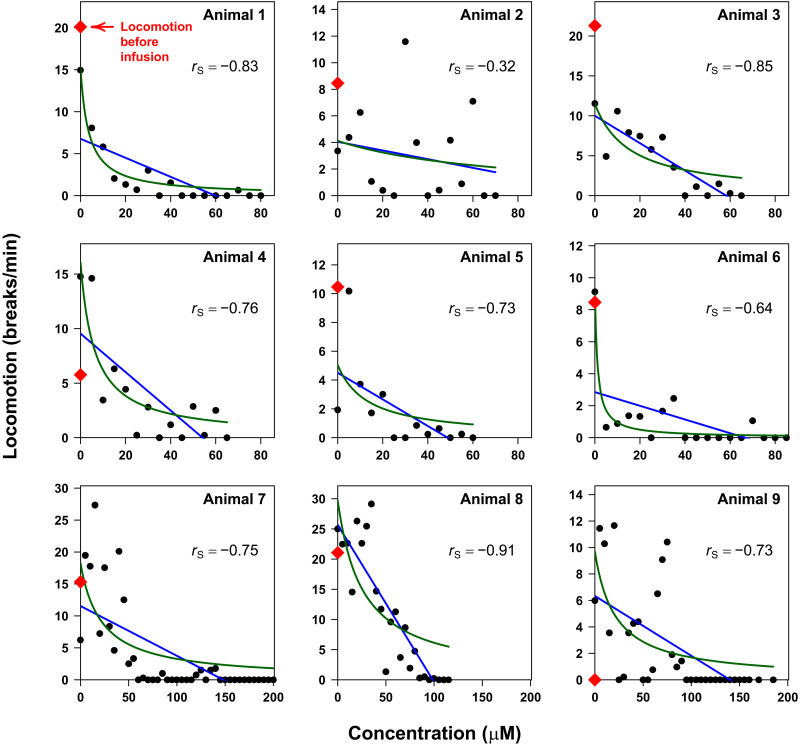
Individual subject correlations between neuropharmacokinetics and locomotor behavior during procaine exposure. Locomotion decreases with increasing intracranial concentrations of the anesthetic procaine. To see this, here, we have binned the locomotion data by procaine concentration (in 5 μM increments) after the start of the infusion to determine the mean number of beam breaks per minute spent at that concentration. Doing so, we observe negative Spearman’s rank correlation estimates, *r*_s_, between the extent of locomotion and drug concentration (bin midpoint) for all nine of our study animals. We have also fit these data to linear (blue curves) and hyperbolic (green curves) models; see text for details. For fitted parameter estimates, see Table 2.

The excellent time resolution and high precision of EAB sensors also render it possible to identify relations between drug pharmacokinetics and drug-induced changes in behavior (i.e., pharmacodynamics) at the level of individual animals with generally good statistical confidence (Fig. 4). As systemic procaine acts as a general anesthetic, the behavioral response we used was locomotion, which we measured using a locomotor chamber equipped with arrays of infrared beams. As expected, locomotor behavior falls for all animals as intracranial procaine levels rise and are then recovered as the drug is metabolized and its concentration falls (Figs. 2 and 3). To put these relationships on a more quantitative footing, we first estimated the Spearman’s rank correlation coefficient (*r*_S_), which provides a measure of the monotonicity of a relationship, between intracranial procaine and locomotion in the *X*-*Y* plane. As expected, this estimated correlation is negative (higher procaine equates to reduced locomotion) for all nine animals (Table 2) and is statistically significant for eight of them. Notably, the single animal for which this correlation is not significant is among the animals that exhibited the least locomotion before infusion, suggesting, perhaps not unreasonably, that the correlation between locomotion and intracranial procaine levels fails if the animal is inactive even before the application of the anesthetic.

**Table 2.** Modeling of locomotion during procaine exposure in individual subjects. *r*_p_ denotes the estimate of the true Pearson’s correlation coefficient ρ_P_. *r*_s_ denotes the estimate of the true Spearman’s correlation coefficient ρ_S_.

Animal no.	Mean locomotion before infusion (breaks per minute)	*r*_s_	Linear model				Hyperbolic model		
			Intercept (breaks per minute)	Slope	*r*_p_	BIC*	Intercept (breaks per minute)	*K*_d_ (μM)	BIC*
**Lateral ventricle, 80 mg/kg dosing**									
**1**	20.1	−0.83†	7 ± 3	−0.11 ± 0.06	−0.71†	90.6	15 ± 2	3.6 ± 1.4	**58.5**
**2**	8.5	−0.32	4 ± 3	−0.03 ± 0.08	−0.21	**85.8**	4 ± 4	70 ± 290	**85.9**
**3**	21.3	−0.85†	10 ± 2	−0.17 ± 0.05	−0.88†	**65.1**	12 ± 5	16 ± 14	72.4
**4**	5.8	−0.76†	10 ± 4	−0.18 ± 0.09	−0.74†	80.6	16 ± 5	6 ± 4	**70.2**
**5**	10.5	−0.73†	5 ± 2	−0.09 ± 0.07	−0.64†	**63.7**	5 ± 4	13 ± 27	**65.3**
**6**	8.5	−0.64†	3 ± 2	−0.04 ± 0.03	−0.53†	80.4	9 ± 2	1.2 ± 0.9	**49.3**
**Lateral ventricle, 160 mg/kg dosing**									
**7**	15.3	−0.75†	12 ± 3	−0.08 ± 0.03	−0.67†	**261.2**	18 ± 8	22 ± 20	**259.7**
**8**	21.1	−0.91†	26 ± 4	−0.26 ± 0.06	−0.88†	**152.6**	30 ± 10	26 ± 20	167.4
**9**	0	−0.73†	6 ± 2	−0.05 ± 0.02	−0.62†	**186.2**	10 ± 5	21 ± 22	**186.2**

*Bolded text denote models that the BIC indicates are preferred.

†Denotes estimated correlations are significantly different from 0 at a significance level of α = 0.05

Our use of the Spearman’s correlation to analyze our data does not assume any specific mathematical relationship (other than monotonicity) between the intracranial concentration of the drug and the resulting behavior. In contrast, the more widely used Pearson’s correlation coefficient assumes that the relationship between the two is linear. Using this approach, we once again find that the estimated correlation coefficient, *r*_P_, associated with the relationship between intracranial drug levels and *X*-*Y* plane locomotion is negative for all nine animals (Fig. 4, blue curves) and statistically significant for eight of them (Table 2; see also figs. S7 and S8 and tables S1 and S2 for the equivalent studies using other measures of locomotion). The linear relationship assumed by the Pearson correlation, however, is likely naive. For example, if anesthesia arises due to the binding of procaine to a specific, saturable biological target (such as for, e.g., a neurotransmitter receptor), then the effect might scale hyperbolically (i.e., follow a “Langmuir isotherm”) rather than linearly with drug concentration. To test this, we fitted all nine datasets to hyperbolic relationships (Fig. 4, green curves) and then used the Bayesian Information Criteria (BIC) (*39*) to judge whether this model is statistically preferred over the linear model. We find that, using an established “rule of thumb” (*40*) that two models whose BICs fall within 2 units of one another describe the data equally as well, the hyperbolic model is preferred (has the smallest BIC value) or matches the linear model for seven of the nine animals we characterized and produces estimates of the receptor’s *K*_d_ in the low micromolar range (Table 2). This said, we recognize that even a hyperbolic model may be a naively simple description of the complex physiological processes by which rising procaine concentrations ultimately produce anesthesia. The model, for example, assumes that the degree to which locomotion is reduced is simply proportional to fractional occupancy of the relevant receptor that is bound at a given drug concentration.

**Fig. 5.**
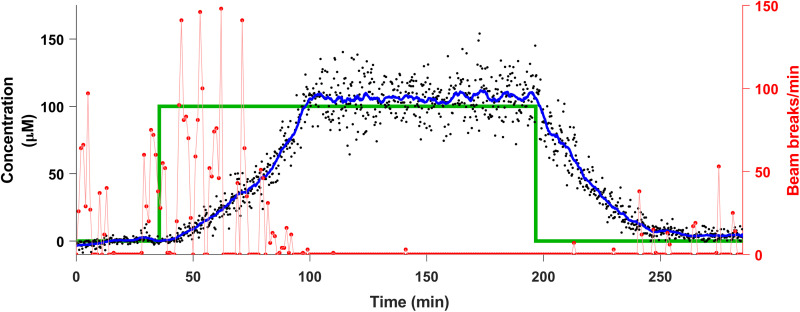
Closed-loop control over in-brain procaine control. The real-time concentration information provided by EAB sensors enables closed-loop feedback control over in vivo drug concentrations. Here, for example, we have used a simple PID feedback control algorithm to control intracranial procaine levels via the regulated intravenous delivery of the drug. The PID controller uses past and current measurements to determine the injection rate required to optimally achieve the desired concentration. We present the targeted concentration levels (green lines) together with the measured procaine concentrations (black dots) and a rolling 6.5-min average (blue curve) of the latter. Also shown are the animal’s *X*-*Y* plane locomotion during the experiment (red curve). The concentration data were Hampel filtered to remove noise artifacts in their presentation; for the raw data, see fig. S9.

The concentration information provided by EAB sensors is not only continuous but also real time, providing opportunities to perform closed-loop feedback control over in vivo drug levels. Previously, we have used a proportional-integral-derivative (PID) feedback controller to achieve and maintain specific, desired plasma drug concentrations (*19*, *20*). Here, we have extended this capability to the control of CSF drug levels using our new intracranial EAB sensor. To do so required that we overcome a new challenge. Specifically, because the measurements are collected intracranially while the drug is injected intravenously, the controller needs to account for nontrivial drug transport delays, requiring careful tuning of the controller. We do not, however, know the exact pharmacokinetics of any specific animal before beginning the experiment. To reduce this problem, we tuned our controller using pharmacokinetic data collected during our continuous infusion experiments (Figs. 2 and 3). With the resulting control, we achieved a targeted, 100 μM procaine concentration about 1 hour after the initiation of control, which we then held for more than 1.5 hours. During the latter period, the root mean square deviation from the set point concentration was just 15 μM, illustrating the high degree of control this approach can achieve. As expected, we see a significant decrease in locomotion during the period of control (Fig. 5). After the control was released, we observe a steady loss of procaine (with a *t*_1/2_ of approximately 20 min) and increasing levels of locomotion.

## DISCUSSION

Here, we have adapted EAB sensors to the problem of monitoring and controlling the concentration of a psychoactive drug in situ in brain ventricles and tissues of live rats in experiments that were performed in parallel with simultaneous observations of the resulting behavioral (locomotor) responses. The resulting measurements achieved time resolution of 10 to 11 s in experiments that spanned hours and enabled the determination of high-accuracy, single-animal, pharmacokinetic profiles. The resulting measurements are also real time and thus provide a means of not only measuring intracranial drug levels but also using closed-loop control systems to actively maintain drug concentrations at an arbitrarily defined level for a prolonged period of time.

The EAB approach described here represents a significant advance in our ability to monitor and control brain chemistry. We are aware of only three prior reports of drug measurements being performed in situ (as opposed to ex vivo) in the brains of behaving animals, none of which were real time and all three of which required averaging over multiple animals to achieve statistical significance. In two of these, Rocchitta *et al.* (*41*, *42*) used an enzymatic alcohol sensor to measure ethanol in the brains of awake, behaving rats. In the third, Taylor *et al.* (*43*) used a sensor similar to that described here to monitor cocaine in the brains of anesthetized rats after intravenous dosing (2 mg/kg), but they had to average over four sensors placed in the brains of two animals. That study reports peak in-brain cocaine concentrations of 15 μM, a value exceeding by several-fold the peak levels measured in prior microdialysis studies using similar drug doses (*4*, *41*, *42*, *44*–*48*).

In vivo EAB sensor measurements represent a vast improvement in the time resolution with which drugs can be measured at specific sites in the brain. For example, the measurements presented here represent an order of magnitude improvement in time resolution over the most highly time-resolved microdialysis studies we have found of intracranial drug concentrations [i.e., 1-min sampling intervals with ~1-min equilibration (*4*)] and a two orders of magnitude improvement over typical microdialysis studies of drugs in the brain [i.e., 10- to 20-min sampling intervals as reviewed in (*17*)]. We believe that this vastly improved resolution, which concomitantly comes with a vastly improved ability to estimate key pharmacokinetic profiles for each subject, will improve our understanding of pharmacology and its effects on behavior. For example, because of the difficulty of measuring intracranial drug concentrations with good precision (much less in real time), current experimental approaches in neuropsychopharmacology almost invariably focus on dose-controlled experiments in drug-induced behavioral perturbations, which are then correlated with total drug dose rather than the concentration (much less the rate of change of concentration) of the drug in the brain. While dose-controlled approaches have long highlighted individual differences in animals’ behavioral responses, they simply cannot reveal the effects of individual differences in animal neuropharmacokinetics. The high-resolution, high-precision, in situ concentration measurements provided by EAB sensors, in contrast, offer opportunities to instead correlate behaviors with the in-brain drug levels (and time-resolved changes in these levels) that are driving them. Given this, it is perhaps not unexpected that, here, EAB sensors have enabled the identification of statistically significant correlations (*r*_P_ and *r*_S_ as great as −0.88 and −0.91, respectively) between intracranial drug levels and behavior (locomotor function) within individual animals.

Moving beyond mere measurements of intracranial drug levels, the EAB sensor–enabled ability to control drug levels in the CSF provides further, likely even more powerful routes to overcoming the shortcomings of prior experimental approaches in neuropsychopharmacology to account for concentration-based factors. Applying EAB-driven feedback control, for example, researchers will be able to precisely manipulate the neuropharmacokinetics of drugs across individual animals, as has been shown with plasma pharmacokinetics (*19*), so as to quantitatively elucidate the relationships between neuropharmacokinetics and behavior. For example, by providing a means of investigating, in an entirely controlled manner, the role that individual pharmacokinetics play in the manifestation and maintenance of addiction, this approach could prove a powerful tool for addiction research. Similarly, the ability to hold drug concentrations static at any desired level could be used to examine neuroadaptive and behavioral responses.

## MATERIALS AND METHODS

### Chemical reagents and materials

All reagents were purchased from Sigma-Aldrich (St. Louis, MO) and used as received. The synthesis of the high-performance liquid chromatography–purified, reporter-and-thiol–modified, procaine-binding aptamer was obtained from Biosearch Technologies (Novato, CA), dissolved in deionized water to 100 μM, aliquoted into separate tubes, and stored at −20°C until used.

### Sensor fabrication and in vitro measurements

The in-brain “probes” we use consist of three parts: the sensor itself (Fig. 1A), an indwelling cannula (Fig. 1B), and a flexible leash, all of which were custom-constructed on the basis of our design by Plastics One (Roanoke, VA). The indwelling cannula was constructed from a 19-gauge stainless steel cannula (2 mm length below pedestal) with a plastic screw-top connector (total length of 13 mm). The sensor was constructed from a 22-gauge threaded stainless steel cannula (15 mm length below pedestal), which served as the counter/pseudo-reference electrode, filled with Teflon-coated gold wire (76 μm in diameter, 3 mm exposed, active sensor length, 19 mm in total length), and surrounded by a plastic screw-top connector with two ports for electrode pins. The electrode leash (50 cm) was built to contain male and female ends for electrode pins on opposite sides and is protected with a stainless-steel mesh exterior.

We first validated the EAB sensor in vitro using devices fabricated on 3-mm-diameter gold wire electrodes, which we tested in undiluted bovine CSF (BioIVT, Westbury, NY) to ensure that they achieved physiologically relevant detection limits and selectivity (i.e., that they reject any endogenous molecules in the CSF). To fabricate sensors on these electrodes, we first cleaned them by electrochemically with the following protocol: (i) 900 cycles between −1 and −1.6 V in a solution of 0.5 M NaOH at 1 V s^−1^ to remove any residual thiol/organic contaminants on the electrode surface and (ii) pulsed between 0 and 2 V for at least 16,000 cycles with a pulse length of 20 ms in 0.5 M H_2_SO_4_ to increase the electrode roughness. To prepare the aptamer for attachment to the gold surface, we first reduced 4 μl of a 100 μM solution of the appropriate thiol and methylene blue–modified oligonucleotide (5′–thiol–C6–PO_4_–AGAC AAG GAA AAT CCT TCA ACG AAG TGG GTCG–PO_4_–methylene blue–3′) using 4 μl of 10 mM of tris(2-carboxyethyl) phosphine hydrochloride for 1 hour at 25°C. We then diluted the solution to 500 nM with 1× phosphate-buffered saline. We then submerged freshly cleaned electrodes in this solution for 1 hour before moving them to a 10 mM 6-mercaptohexanol solution overnight to form a self-assembled monolayer.

Our custom in-brain probes consist of two major parts—an electrode leash and an implantable gold/stainless-steel electrode pair (P1, Roanoke, VA). The former (50 cm length) is composed of two copper wires encased in a braided stainless-steel mesh leash. At each end of the leash are two gold-coated copper pins (1 mm apart) that emerge from a plastic casing and a screw-on plastic collar (8 mm length). One end of this is attached to the implanted electrodes and the other to a two-channel swivel commutator. Before using the electrode pair, we cleaned the gold electrode and applied the DNA aptamer and self-assembling monolayer to it as described above.

To optimize the electrochemical parameters used in sensor interrogation, we conducted a 50-point titration of our sensors at eight different square-wave frequencies in vitro in undiluted bovine CSF at 37°C and determined the “signal-on” (150 Hz) and “signal-off” (30 Hz) frequencies with the largest relative changes in current. Using serial voltammetric measurements performed at these frequencies, we corrected the baseline drift seen in vivo using “kinetic differential measurements” [KDM; detailed in (*49*)]. To convert KDM signals into drug concentrations, we fitted the in vitro titration data to the equation: $y=B_{\mathrm{MAX}}\times \frac{\left[\mathrm{target}\right]}{K_{d}+[\mathrm{target}]}$, where *y* is the KDM signal, *B*_MAX_ is the signal change seen at saturating target concentrations, [target] is the drug concentration, and *K*_d_ is the *K*_d_ of the aptamer.

### Animal housing and care

Adult female (225 to 250 g) and male (275 to 300 g) Sprague-Dawley rats obtained with a jugular catheter already emplaced (Charles River Laboratories, Hollister, CA); the chronic indwelling jugular catheters enabled rapid drug delivery. The rats were pair-housed in a standard light cycle room (08:00 on, 20:00 off) and allowed ad libitum access to food and water. Daily monitoring was performed by trained animal technicians to ensure animal health and behavioral responsiveness. This monitoring uncovered no evidence that cannula and sensor implantation cause harm. All animals continued to gain weight after implantation of the cannula through to the completion of our experiments. The housing and care of all rats were conducted in accordance with the guidelines set forth by the “Guide for the Care and Use of Laboratory Rats,” 8th edition, (*50*), and all experiments were approved by the University of California, Santa Barbara Institutional Animal Care and Use Committee.

### Surgery

We induced anesthesia before surgery using 4% isoflurane gas (Medline, Northfield, IL) and maintained it using 2 to 3% for the remainder of surgical procedures. After inducing anesthesia, we shaved the head of the rats and disinfected the surgical site with 10% betadine solution and 70% isopropanol before injecting the local anesthetic lidocaine (2 mg/kg subcutaneously) into each incision site. Temperature, heart rate, SPO_2_ (specific peripheral oxygen concentration), and respiration rate were recorded and maintained within veterinary recommended levels during surgical procedures. Next, we implanted a permanent 19-gauge stainless-steel guide cannula (Plastics One, Roanoke, VA) using stereotaxic coordinates aimed at the lateral ventricle [anteroposterior (AP) = −0.48, mediolateral (ML) = +1.6, dorsoventral (DV) = −1] or the hippocampus (AP = −4.8, ML = +5.1, DV = −1), which was affixed to the skull with metal screws and covered in dental cement. Once the cannula was implanted, a removable 1.2-cm-long 22-gauge stainless-steel obturator (the length of which places its tip at the same depth as the probe tip) was placed into the cannula. After surgery, rats are handled daily, and catheters are flushed to ensure patency as well as general health of the rat with a minimum of 7 days before experimentation. Following the experiment, we removed the brains from the rats and performed histology to confirm sensor placement (see below).

### In vivo measurements

Before commencing measurements, we weighed each rat (mean ± SEM weights for females, 290 ± 13 g, and for males, 418 ± 6 g) and induced anesthesia using 3% isoflurane gas and maintained anesthesia as we first removed the obdurator, inserted the sensor through the cannula (~10 to 15 s), and then affixed the sensor in place by the screw fittings on the surgically implanted 19-gauge guide cannula. We then placed the rat into a Kinder Scientific Motor Monitor system (Fig. 1E), allowed them to recover from anesthesia, and began recording locomotion via the supplied Motor Monitor software. We commenced EAB measurements using square-wave voltammetry on a CH1208C mini-potentiostat, alternating between square-wave frequencies of 30 Hz (swept at 0.02 V/s) and 150 Hz (swept at 0.15 V/s), both at an amplitude of 25 mV and over a voltage window of approximately 0.1 to −0.27 V (relative to the stainless-steel pseudo-reference). To visualize our measurements in real time, we used Software for the Analysis and Continuous Monitoring of Electrochemical Systems (SACMES), an open-source Python script previously reported by our laboratories (*51*).

### Feedback control

We use a PID feedback controller to achieve the targeted level of procaine. PID controllers have three tunable parameters corresponding to their namesake processes. We use the data from our infusion experiments (Figs. 2 and 3) to inform on the tuning parameter values that would result in acceptable performance had we conducted feedback control on all of these animals. We use these tuned values for the PID controller that we used in the experiment shown (Fig. 5). Because our tuning is based on the pharmacokinetics of animals other than the animal we are actually experimenting on, we also implemented a fail-safe mechanism to avoid possible overshooting (and overdosing). This mechanism stops the pump from injecting the drug if the intracranial concentration exceeds 110% of the targeted value, and it does not reactivate the PID controller unless the concentration level falls below 105% of the targeted value.

### Histology

To ensure proper sensor placement, we euthanized the rats at the end of the experiment using an intravenous injection of 0.1 ml of Somnasol (Covetrus, Chicago, IL) before performing histology. To do so, we decapitated the animals with a guillotine (Braintree Scientific, Braintree, MA), collected their brains in 10% formalin, incubated them in this for 1 week, and then transferred them to 30% sucrose solution for 3 days. Following this, we blocked the brains at the cerebellum, flash-frozen them over dry ice for 1 min, mounted them on a stand, and sliced them into 40-μm sections using a cryostat. These were then Nissl-stained and examined on a Nikon E800 microscope equipped with a Hamamatsu Orca camera to verify cannula placements (fig. S5 for example lateral ventricle and hippocampus placement).

### Statistical analyses

The high-frequency measurements provided by EAB sensors vastly increase the number of concentration measurements within any fixed time interval (e.g., by two orders of magnitude over typical microdialysis studies) of in-brain drug concentrations in individual animals. The resulting high number of measurements for each animal enables us to perform within-subject statistical analyses on individual animals, which we believe is effectively unprecedented. Thus, motivated, we explored the potential association between drugs and locomotion behavior, after binning the data collected after the start of infusion into different concentration levels of drug present in the tissue, adjusted for amount of time within that concentration bin. We binned concentrations into 5 μM increments (−2.5 to 2.5 μM, 2.5 to 7.5 μM, etc.; with inclusivity on the upper bound), labeled by their midpoints. Concentration measurements falling below 0 μM (which arise due to sensor noise) were grouped into the 0 μM bin. We considered data only after the start of infusion. Within each concentration bin, we calculated the sum of all locomotion counts divided by the total amount of time (in minutes) spent in that bin; effectively returning a new measurement (breaks per minute) average for that concentration bin.

We investigated the relationship between procaine concentration bins and beam breaks per minute using two different correlation measures: those being the Pearson’s correlation coefficient and Spearman’s rank correlation coefficient. Pearson’s correlation coefficient measures the strength and direction of a linear association (here being the concentration bin midpoints and the breaks per minute)(*52*). Spearman’s rank correlation coefficient measures the strength and direction of a monotonic association between two variables (*53*). Note that the Spearman’s rank correlation is the Pearson’s correlation between the ranked data. Table 1 presents estimated Spearman’s rank and Pearson’s correlation coefficients for each animal, along with whether we rejected the null hypothesis of 0 correlation (for Pearson’s) or lack of monotonicity (for Spearman’s) at a significance level of α = 0.05 (i.e., whether a *P* value less than significance level α resulted in a rejection of the null hypothesis). Results were obtained using the cor.test function in R (*54*). We present results for both correlation coefficients in this paper, although Spearman’s rank correlation coefficient is more appropriate due to the monotonic yet curved pattern data exhibit in a majority of the panels in Fig. 5. Simple linear regression lines are included in Fig. 5 as a visual reference for the Pearson’s correlation estimate. A “*” in Table 1 indicates rejection of the null hypothesis that either (i) no linear relationship (Pearson’s) or (ii) no monotonic relationship (Spearman’s rank) between the binned concentration midpoints and the breaks per minute (i.e., *H*_0_: ρ*_p_ =* 0 or *H*_0_: ρ*_s_ =* 0 as in Table 1 notation). Because of ties when ranking the data for estimating Spearman’s rank correlation, approximate *P* values were calculated and used to determine significance (*53*).

The hyperbolic function, *h*, used to model the binned concentration midpoints and break per minute data (results of which are seen in Table 2 and Fig. 4) is

$$h(c)=M_{0}\left(\frac{K_{d}}{c+K_{d}}\right)$$

where *c* is the functional input of concentration bin midpoint, *K*_d_ is the receptor’s dissociation parameter, and *M*_0_ is the intercept parameter of the model—both of which are the unknown parameters of the model to be estimated.

## Notes

View/request a protocol for this paper from *Bio-protocol*.

## References

[1] J. I. Javaid, M. W. Fischman, C. R. Schuster, H. Dekirmenjian, J. M. Davis, Cocaine plasma concentration: Relation to physiological and subjective effects in humans. Science 202, 227–228 (1978). 694530 10.1126/science.694530

[2] A. R. Jeffcoat, M. Perez-Reyes, J. M. Hill, B. M. Sadler, C. E. Cook, Cocaine disposition in humans after intravenous injection, nasal insufflation (snorting), or smoking. Drug Metab. Dispos. 17, 153–159 (1989). 2565204

[3] J. K. Melichar, M. R. Daglish, D. J. Nutt, Addiction and withdrawal–current views. Curr. Opin. Pharmacol. 1, 84–90 (2001). 11712541 10.1016/s1471-4892(01)00011-x

[4] E.-A. Minogianis, W. M. Shams, O. S. Mabrouk, J. M. T. Wong, W. G. Brake, R. T. Kennedy, P. Souich, A. N. Samaha, Varying the rate of intravenous cocaine infusion influences the temporal dynamics of both drug and dopamine concentrations in the striatum. Eur. J. Neurosci. 50, 2054–2064 (2019). 29757478 10.1111/ejn.13941 PMC6296906

[5] J. E. Usubiaga, F. Moya, J. A. Wikinski, R. Wikinski, L. E. Usubiaga, Relationship between the passage of local anaesthetics across the blood-brain barrier and their effects on the central nervous system. Br. J. Anaesth. 39, 943–947 (1967). 6082561 10.1093/bja/39.12.943

[6] A. Kitamura, T. Okura, K. Higuchi, Y. Deguchi, Cocktail-dosing microdialysis study to simultaneously assess delivery of multiple organic-cationic drugs to the brain. J. Pharm. Sci. 105, 935–940 (2016). 26554532 10.1002/jps.24691

[7] B. Reiter, T. Stimpfl, Quantification of drugs in brain samples. J. Anal. Toxicol. 39, 702–706 (2015). 26232450 10.1093/jat/bkv078

[8] P. K. Garg, S. J. Lokitz, R. Nazih, S. Garg, Biodistribution and radiation dosimetry of ^11^C-nicotine from whole-body pet imaging in humans. J. Nucl. Med. 58, 473–478 (2017). 27660145 10.2967/jnumed.116.180059

[9] W. W. Moses, Fundamental limits of spatial resolution in PET. Nucl. Instrum. Methods Phys. Res. A 648 (Suppl. 1), S236–S240 (2011). 21804677 10.1016/j.nima.2010.11.092 PMC3144741

[10] J. E. Rose, A. G. Mukhin, S. J. Lokitz, T. G. Turkington, J. Herskovic, F. M. Behm, S. Garg, P. K. Garg, Kinetics of brain nicotine accumulation in dependent and nondependent smokers assessed with PET and cigarettes containing ^11^C-nicotine. Proc. Natl. Acad. Sci. U.S.A. 107, 5190–5195 (2010). 20212132 10.1073/pnas.0909184107 PMC2841893

[11] U. Ungerstedt, *Introduction to Intracerebral Microdialysis* (Elsevier, 1991).

[12] V. I. Chefer, A. C. Thompson, A. Zapata, T. S. Shippenberg, Overview of brain microdialysis. Curr. Protoc. Neurosci. CHAPTER 7, Unit7.1 (2009). 10.1002/0471142301.ns0701s47 PMC2953244 19340812

[13] M. Wang, N. D. Hershey, O. S. Mabrouk, R. T. Kennedy, Collection, storage, and electrophoretic analysis of nanoliter microdialysis samples collected from awake animals in vivo. Anal. Bioanal. Chem. 400, 2013–2023 (2011). 21465093 10.1007/s00216-011-4956-9 PMC3107505

[14] C. K. Su, S. C. Hsia, Y. C. Sun, A high-throughput microdialysis-parallel solid phase extraction-inductively coupled plasma mass spectrometry hyphenated system for continuous monitoring of extracellular metal ions in living rat brain. J. Chromatogr. A 1326, 73–79 (2014). 24388243 10.1016/j.chroma.2013.12.047

[15] T. Ngernsutivorakul, D. J. Steyer, A. C. Valenta, R. T. Kennedy, In vivo chemical monitoring at high spatiotemporal resolution using microfabricated sampling probes and droplet-based microfluidics coupled to mass spectrometry. Anal. Chem. 90, 10943–10950 (2018). 30107117 10.1021/acs.analchem.8b02468 PMC6939306

[16] T. Ngernsutivorakul, T. S. White, R. T. Kennedy, Microfabricated probes for studying brain chemistry: A review. ChemPhysChem 19, 1128–1142 (2018). 29405568 10.1002/cphc.201701180 PMC6996029

[17] H. Yang, A. B. Thompson, B. J. McIntosh, S. C. Altieri, A. M. Andrews, Physiologically relevant changes in serotonin resolved by fast microdialysis. ACS Chem. Nerosci. 4, 790–798 (2013). 10.1021/cn400072f PMC3656759 23614776

[18] R. A. Saylor, S. M. Lunte, A review of microdialysis coupled to microchip electrophoresis for monitoring biological events. J. Chromatogr. A 1382, 48–64 (2015). 25637011 10.1016/j.chroma.2014.12.086 PMC4403801

[19] N. Arroyo-Currás, G. Ortega, D. A. Copp, K. L. Ploense, Z. A. Plaxco, T. E. Kippin, J. P. Hespanha, K. W. Plaxco, High-precision control of plasma drug levels using feedback-controlled dosing. ACS Pharmacol. Transl. Sci. 1, 110–118 (2018). 32219207 10.1021/acsptsci.8b00033 PMC7088981

[20] P. Dauphin-Ducharme, K. Yang, N. Arroyo-Currás, K. L. Ploense, Y. Zhang, J. Gerson, M. Kurnik, T. E. Kippin, M. N. Stojanovic, K. W. Plaxco, Electrochemical aptamer-based sensors for improved therapeutic drug monitoring and high-precision, feedback-controlled drug delivery. ACS Sens. 4, 2832–2837 (2019). 31556293 10.1021/acssensors.9b01616 PMC6886665

[21] J. P. Newman, M.-f. Fong, D. C. Millard, C. J. Whitmire, G. B. Stanley, S. M. Potter, Optogenetic feedback control of neural activity. eLife 4, e07192 (2015). 26140329 10.7554/eLife.07192 PMC4490717

[22] M. Prsa, G. L. Galiñanes, D. Huber, Rapid integration of artificial sensory feedback during operant conditioning of motor cortex neurons. Neuron 93, 929–939.e6 (2017). 28231470 10.1016/j.neuron.2017.01.023 PMC5330804

[23] M. Shin, Y. Wang, J. R. Borgus, B. J. Venton, Electrochemistry at the synapse. Annu. Rev. Anal. Chem. 12, 297–321 (2019). 10.1146/annurev-anchem-061318-115434 PMC6989097 30707593

[24] R. B. Keithley, P. Takmakov, E. S. Bucher, A. M. Belle, C. A. Owesson-White, J. Park, R. M. Wightman, Higher sensitivity dopamine measurements with faster-scan cyclic voltammetry. Anal. Chem. 83, 3563–3571 (2011). 21473572 10.1021/ac200143v PMC3089759

[25] S. Qin, M. Van Der Zeyden, W. H. Oldenziel, T. I. F. H. Cremers, B. H. C. Westerink, Microsensors for in vivo measurement of glutamate in brain tissue. Sensors 8, 6860–6884 (2008). 27873904 10.3390/s8116860 PMC3787420

[26] N. Arroyo-Currás, J. Somerson, P. A. Vieira, K. L. Ploense, T. E. Kippin, K. W. Plaxco, Real-time measurement of small molecules directly in awake, ambulatory animals. Proc. Natl. Acad. Sci. U.S.A. 114, 645–650 (2017). 28069939 10.1073/pnas.1613458114 PMC5278471

[27] A. Idili, N. Arroyo-Currás, K. L. Ploense, A. T. Csordas, M. Kuwahara, T. E. Kippin, K. W. Plaxco, Seconds-resolved pharmacokinetic measurements of the chemotherapeutic irinotecan in situ in the living body. Chem. Sci. 10, 8164–8170 (2019). 31673321 10.1039/c9sc01495k PMC6788505

[28] P. H. Azimi, Concentrations of procaine and aqueous penicillin in the cerebrospinal fluid of infants treated for congenital syphilis. J. Pediatr. 124, 649–653 (1994). 8151486 10.1016/s0022-3476(05)83151-8

[29] W. F. Tu, G. F. Lin, J. F. Shen, J. G. Xu, Changes in erythrocyte membrane ATPases and plasma lipid peroxides in upper abdominal surgery under intravenous procaine-balanced anesthesia. World J. Gastroenterol. 4, 430–433 (1998). 11819338 10.3748/wjg.v4.i5.430 PMC4767744

[30] G. Veneziano, J. D. Tobias, Chloroprocaine for epidural anesthesia in infants and children. Pediatr. Anesth. 27, 581–590 (2017). 10.1111/pan.13134 28321983

[31] L. Winter, Intravenous procaine infusions in the postoperative period. Ann. Surg. 132, 143–146 (1950). 15426198 PMC1616633

[32] A. A. Shoara, O. Reinstein, O. A. Borhani, T. R. Martin, S. Slavkovic, Z. R. Churcher, P. E. Johnson, Development of a thermal-stable structure-switching cocaine-binding aptamer. Biochimie 145, 137–144 (2018). 28838608 10.1016/j.biochi.2017.08.010

[33] A. B. Seifen, A. A. Ferrari, E. E. Seifen, D. S. Thompson, J. Chapman, Pharmacokinetics of intravenous procaine infusion in humans. Anesth. Analg. 58, 382–386 (1979). 573562 10.1213/00000539-197909000-00007

[34] W. W. Qin, Simultaneous determination of procaine, lidocaine, ropivacaine, tetracaine and bupivacaine in human plasma by high-performance liquid chromatography. J. Chromatogr. B Analyt. Technol. Biomed. Life Sci. 878, 1185–1189 (2010). 10.1016/j.jchromb.2010.03.003 20356808

[35] A. M. Downs, J. Gerson, M. N. Hossain, K. Ploense, M. Pham, H. B. Kraatz, T. Kippin, K. W. Plaxco, Nanoporous gold for the miniaturization of in vivo electrochemical aptamer-based sensors. ACS Sens. 6, 2299–2306 (2021). 34038076 10.1021/acssensors.1c00354 PMC12045558

[36] V. Hanušová, P. Tomšík, L. Kriesfalusyová, A. Pakostová, I. Boušová, L. Skálová, In vivo effect of oracin on doxorubicin reduction, biodistribution and efficacy in Ehrlich tumor bearing mice. Pharmacol. Rep. 65, 445–452 (2013). 23744429 10.1016/s1734-1140(13)71020-x

[37] D. Zou, W. Wang, D. Lei, Y. Yin, P. Ren, J. Chen, T. Yin, B. Wang, G. Wang, Y. Wang, Penetration of blood–brain barrier and antitumor activity and nerve repair in glioma by doxorubicin-loaded monosialoganglioside micelles system. Int. J. Nanomed. 12, 4879–4889 (2017). 10.2147/IJN.S138257 PMC5511015 28744122

[38] G. W. Edmonds, Intravenous use of procaine in general anesthesia. JAMA 141, 761 (1949). 10.1001/jama.1949.02910110013005 15391480

[39] G. Schwarz, Estimating the dimension of a model. Ann. Stat. 6, 461–464 (1978).

[40] F. J. Fabozzi, *The Basics of Financial Econometrics* (John Wiley & Sons Inc., 2014).

[41] G. Rocchitta, O. Secchi, M. D. Alvau, R. Migheli, G. Calia, G. Bazzu, D. Farina, M. S. Desole, R. D. O’Neill, P. A. Serra, Development and characterization of an implantable biosensor for telemetric monitoring of ethanol in the brain of freely moving rats. Anal. Chem. 84, 7072–7079 (2012). 22823474 10.1021/ac301253h

[42] G. Rocchitta, A. T. Peana, G. Bazzu, A. Cossu, S. Carta, P. Arrigo, A. Bacciu, R. Migheli, D. Farina, M. Zinellu, E. Acquas, P. A. Serra, Simultaneous wireless and high-resolution detection of nucleus accumbens shell ethanol concentrations and free motion of rats upon voluntary ethanol intake. Alcohol 78, 69–78 (2019). 31029631 10.1016/j.alcohol.2019.04.002

[43] I. M. Taylor, Z. du, E. T. Bigelow, J. R. Eles, A. R. Horner, K. A. Catt, S. G. Weber, B. G. Jamieson, X. T. Cui, Aptamer-functionalized neural recording electrodes for the direct measurement of cocaine in vivo. J. Mater. Chem. B 5, 2445–2458 (2017). 28729901 10.1039/C7TB00095B PMC5512874

[44] S. H. Ahmed, D. Lin, G. F. Koob, L. H. Parsons, Escalation of cocaine self-administration does not depend on altered cocaine-induced nucleus accumbens dopamine levels. J. Neurochem. 86, 102–113 (2003). 12807430 10.1046/j.1471-4159.2003.01833.x

[45] C. W. Bradberry, J. B. Nobiletti, J. D. Elsworth, B. Murphy, P. Jatlow, R. H. Roth, Cocaine and cocaethylene: Microdialysis comparison of brain drug levels and effects on dopamine and serotonin. J. Neurochem. 60, 1429–1435 (1993). 8455033 10.1111/j.1471-4159.1993.tb03305.x

[46] Y. L. Hurd, J. Kehr, U. Ungerstedt, In vivo microdialysis as a technique to monitor drug transport: Correlation of extracellular cocaine levels and dopamine overflow in the rat brain. J. Neurochem. 51, 1314–1316 (1988). 3418351 10.1111/j.1471-4159.1988.tb03103.x

[47] H.-T. Pan, S. Menacherry, J. B. Justice Jr., Differences in the pharmacokinetics of cocaine in naive and cocaine-experienced rats. J. Neurochem. 56, 1299–1306 (1991). 2002342 10.1111/j.1471-4159.1991.tb11425.x

[48] H. O. Pettit, A. J. Pettit, Disposition of cocaine in blood and brain after a single pretreatment. Brain Res. 651, 261–268 (1994). 7922573 10.1016/0006-8993(94)90705-6

[49] B. S. Ferguson, D. A. Hoggarth, D. Maliniak, K. Ploense, R. J. White, N. Woodward, K. Hsieh, A. J. Bonham, M. Eisenstein, T. Kippin, K. W. Plaxco, H. T. Soh, Real-time, aptamer-based tracking of circulating therapeutic agents in living animals. Sci. Transl. Med. 5, 213ra165 (2013). 10.1126/scitranslmed.3007095 PMC4010950 24285484

[50] *IACUC, Guide for the Care and Use of Laboratory Animals: Eighth Edition* (The National Academies Press, 2011). 21595115

[51] S. D. Curtis, K. L. Ploense, M. Kurnik, G. Ortega, C. Parolo, T. E. Kippin, K. W. Plaxco, N. Arroyo-Currás, Open source software for the real-time control, processing, and visualization of high-volume electrochemical data. Anal. Chem. 91, 12321–12328 (2019). 31462040 10.1021/acs.analchem.9b02553 PMC7336365

[52] D. D. Wackerly, W. Mendenhall, R. L. Scheaffer, *Mathematical statistics with applications* (Thomson Brooks/Cole, 2008).

[53] M. Hollander, D. A. Wolfe, E. Chicken, *Nonparametric Statistical Methods*, 3rd Edition|Wiley (John Wiley & Sons Inc., 2014).

[54] R Core Team, R: A language and environemnt for statistical computing (2020); www.R-project.org/.

