# Supplementary material for: High-precision monitoring of and feedback control over drug concentrations in the brains of freely moving rats
Source: Sci Adv. 2023 May 17;9(20):eadg3254. doi: 10.1126/sciadv.adg3254 (PMC10191434; doi:10.1126/sciadv.adg3254)
Supplement: Supplementary file 1 — Figs. S1 to S10 Table S1 and S2 [file sciadv.adg3254_sm.pdf]

Science Advances
AAAS

Supplementary Materials for

High-precision monitoring of and feedback control over drug concentrations in the brains of freely moving rats

Julian Gerson *et al.*

Corresponding author: Tod E. Kippin, kippin@ucsb.edu

Sci. Adv. **9**, eadg3254 (2023)
DOI: 10.1126/sciadv.adg3254

This PDF file includes:

Figs. S1 to S10
Tables S1 and S2

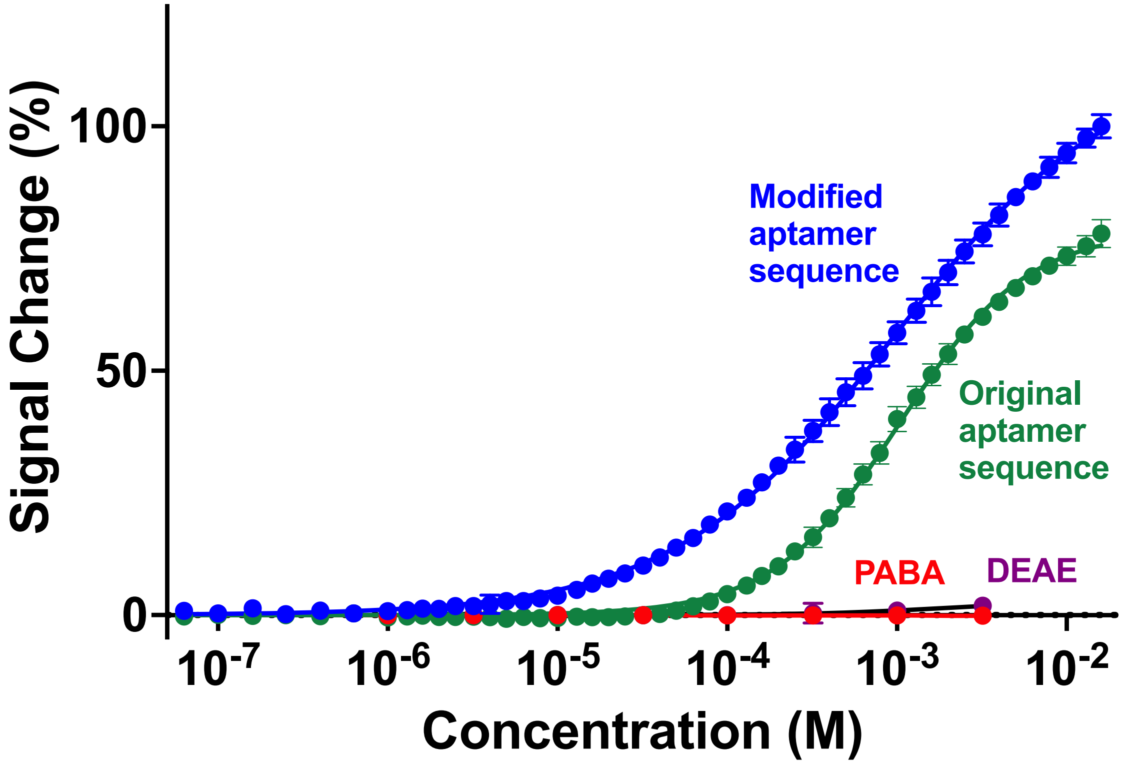

Figure SI1. Calibration Curve for the EAB against Procaine. Binding-induced folding of the aptamer in an EAB sensor leads to an easily measured change in signal that is quantitatively and monotonically related to target concentration. The EAB sensors we have employed here (see methods section for full aptamer sequence) use modifications to a previously reported, cocaine-binding aptamer sequence (32) that improve both signal gain and procaine affinity. The apparent dissociation constant of the new sequence is 960±30 μM (fit using a Langmuir-Hill equation). The new sensor does not, in contrast, detectably respond to either metabolite of procaine, para-amino benzoic acid (PABA) or diethylaminoethanol (DEAE). These calibration curves were collected in undiluted bovine CSF at physiological temperature (37°C). Error Bars represent standard deviation of the mean for independently fabricated sensors.

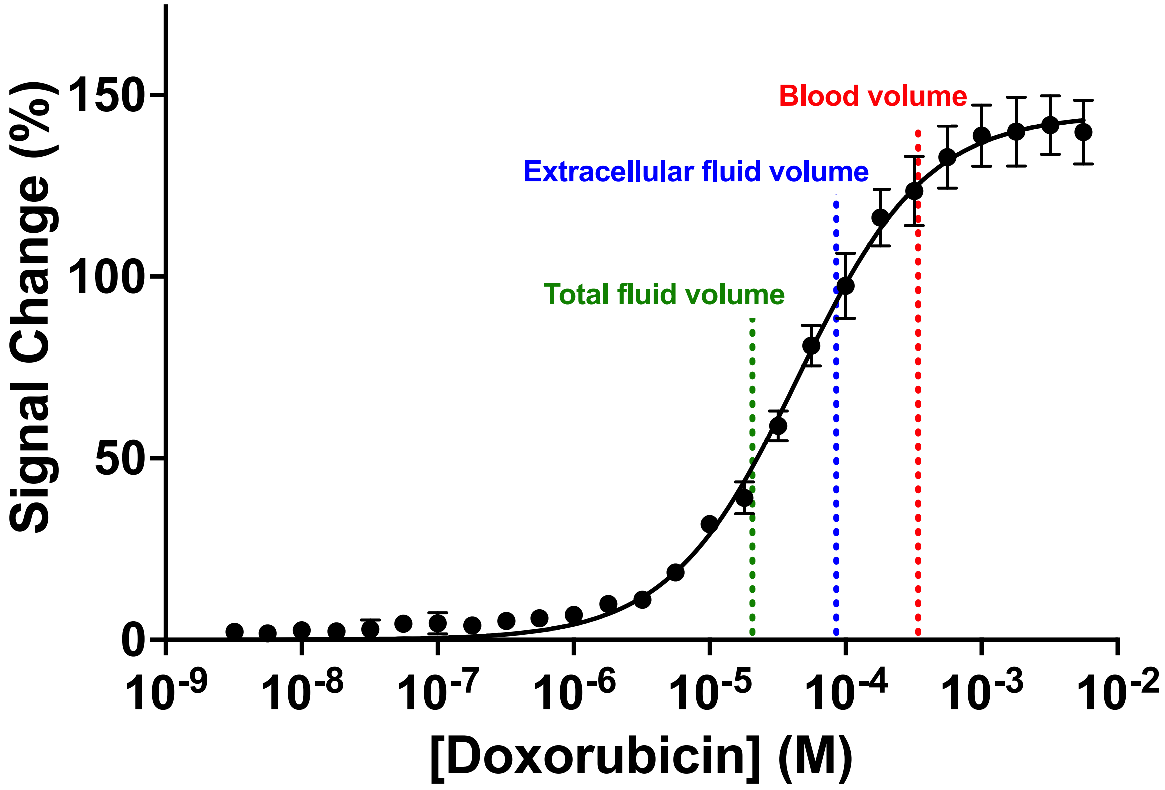

Figure SI2. Calibration Curve for the EAB against Doxorubicin. Shown is the calibration curve of the doxorubicin-detecting EAB sensor we employed as a negative control to detect potential disruption of the blood brain barrier. The chemotherapeutic was dosed intravenously at 12 mg/kg; shown are the resulting concentrations we would expect were that dose distributed evenly throughout the blood, throughout the entire extracellular fluid volume of a rat, or throughout the entire fluid volume of a rat. Even in the latter case, the resulting concentration should be orders of magnitude above the sensor's sub-micromolar limit of detection, and yet we did not observe any significant signal change for the intracranial sensor (Fig. 1G).

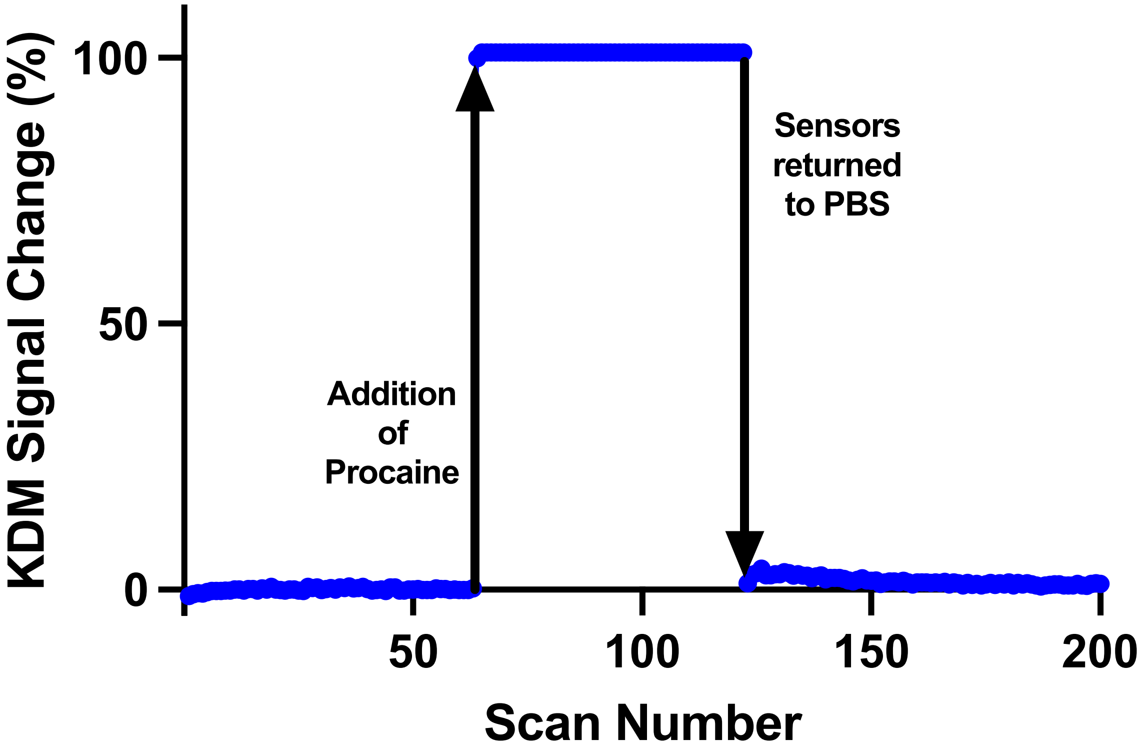

Figure SI3. Temporal Resolution and Reversibility of the EAB against Procaine. The procaine-detecting EAB sensor rapidly responds to the addition of its target, and rapidly reverses upon its removal. Here the sensors (n = 8), which were scanned in 1X PBS at 37˚C, responded to completion within the time required to complete the required pair of square-wave voltammetry scans (~11 s).

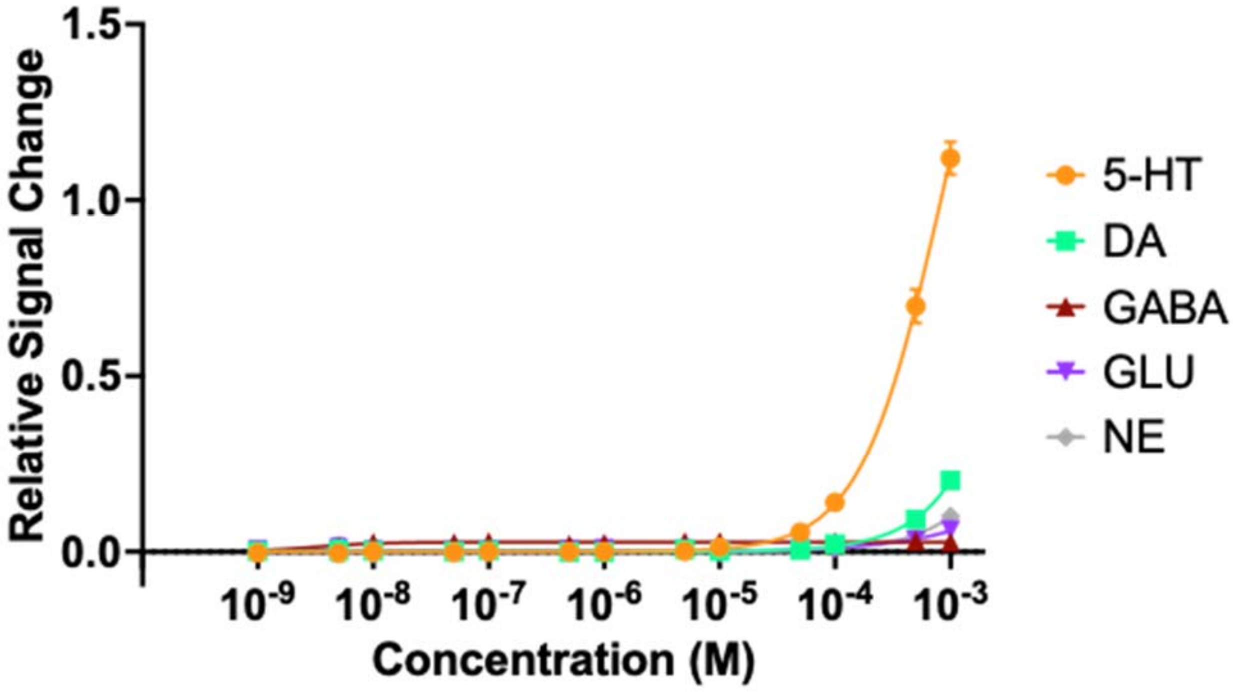

Figure SI4. Common Neurotransmitters do not Induce Signal Change in the EAB against Procaine. The EAB sensors we have employed here do not detectably respond to the major amino acid or amine neurotransmitters within their physiological concentrations. Serotonin (5-HT), dopamine (DA), and norepinephrine (NE) are present in brain at low nM but our sensor shows no response until well above 10 µM and glutamate (GLU) and gamma-aminobutyric acid (GABA) are present in brain at low µM concentration but our sensor shows no response up to the 1 mM level. Experiments performed in undiluted bovine CSF at physiological temperature (37°C).

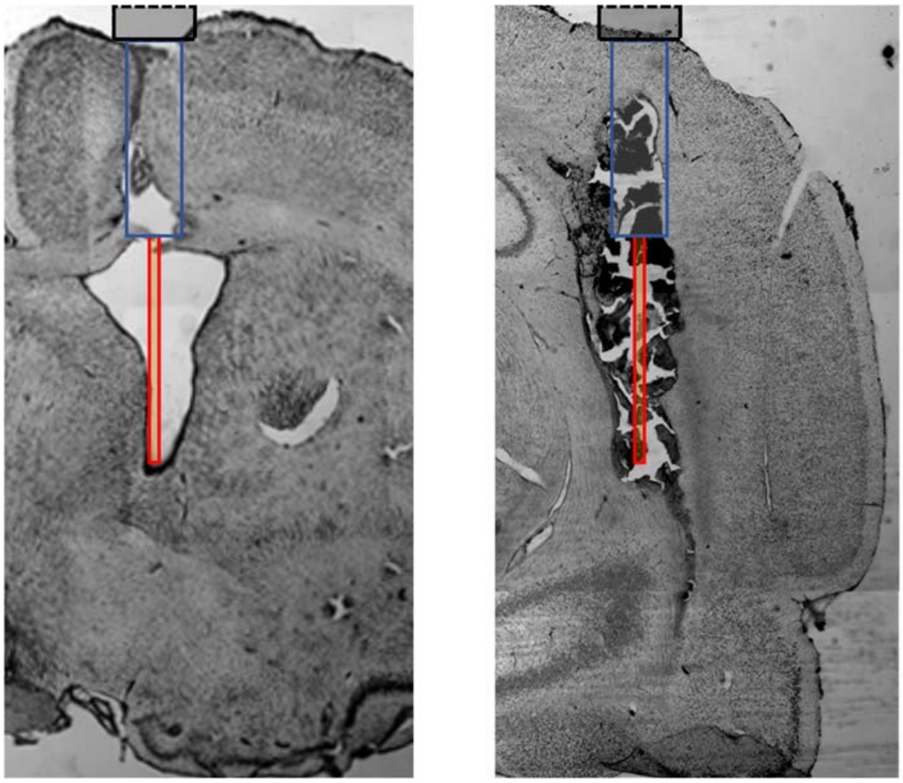

Figure SI5. Histological Confirmation of EAB Placement in Target Region. Examples of histological confirmation of appropriate sensor placement in the lateral ventricle (Left) and hippocampus (Right). To ensure this, and that the working portion of the sensor is entirely in the region of interest, we fix, slice and image all brains post experiment. The left image shows histology from a rat brain with a lateral ventricle placement (as evident by damage above the ventricle but no damage to its walls). The right image shows histology from a rat brain with a hippocampal placement (with evident damage to structure and the ventral most damage indicating location of the bottom tip of the probe). Both images are overlaid with semi-transparent schematic of the sensor with gold representing the working electrode, light gray representing the 22-gague cannula portion of the probe, and dark gray representing the 19-gauge permanent guide cannula.

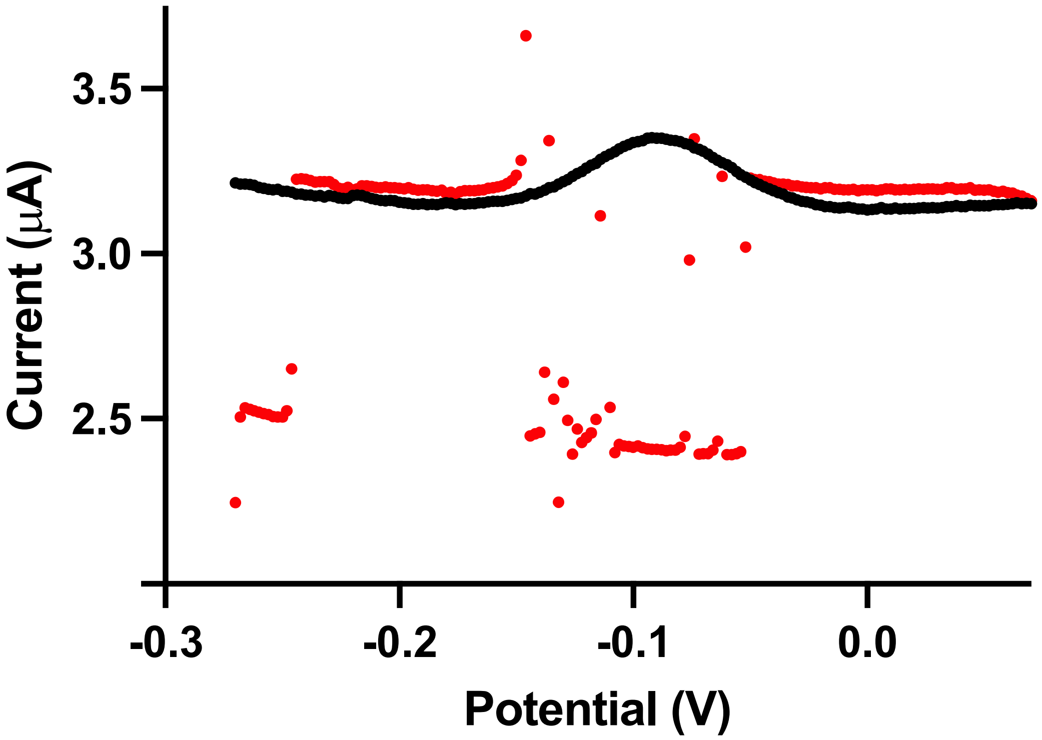

Figure SI6. Mechanical Noise-Induced Disruption of Voltammograms. Example of mechanical noise resulting from the animal striking the probe against the side of the locomotor chamber. A mechanically noisy voltammogram (red) significantly distorts estimates of peak heights compared to voltammograms unaffected by mechanical noise (black). The potential is reported versus a silver/silver chloride reference.

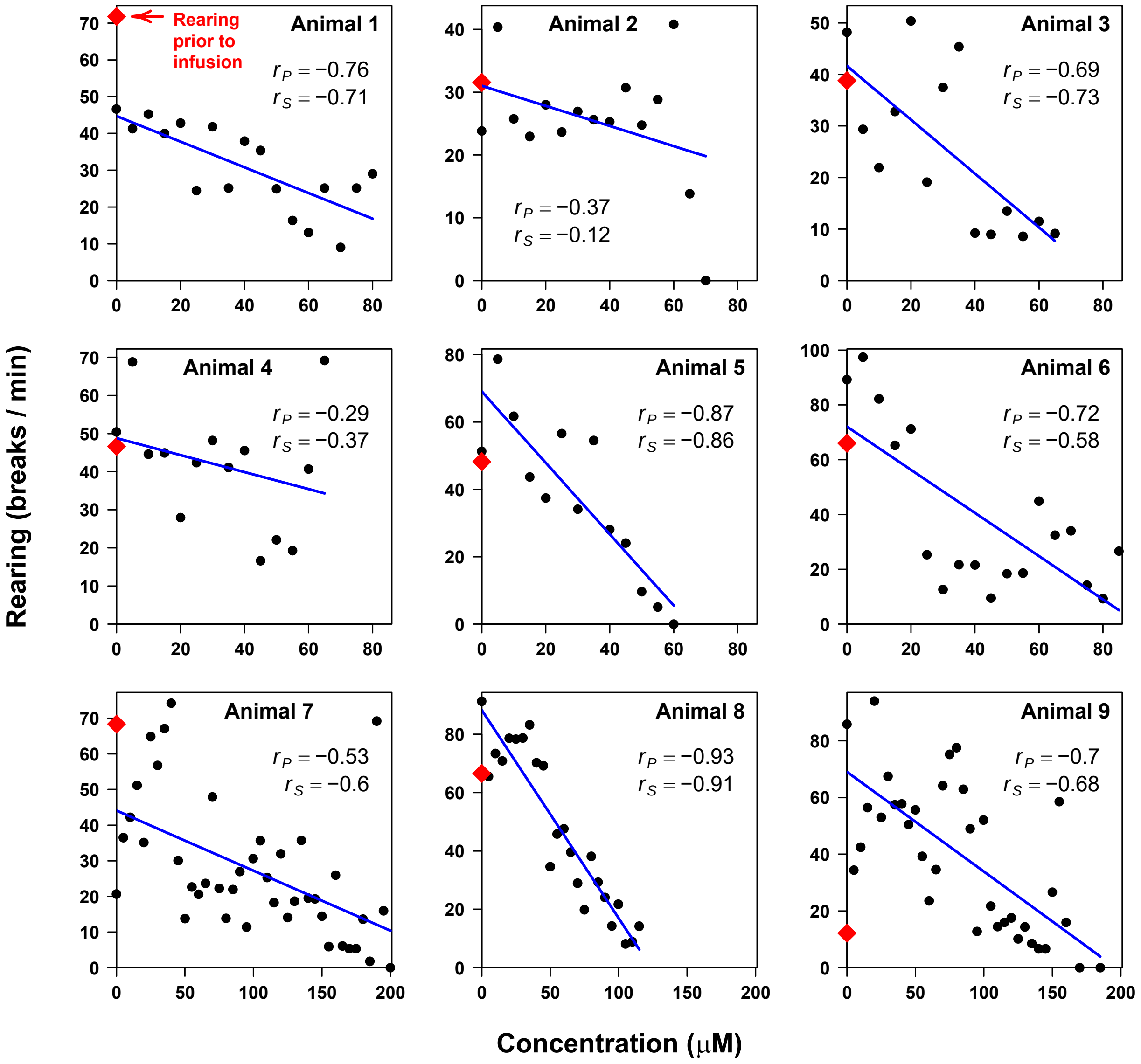

Figure SI7. Individual Subject Correlations between Neuropharmacokinetics and Rearing Behavior during Procaine Exposure. Like X-Y ambulation (Fig. 5), rearing also decreases with increasing intracranial procaine concentrations. Here we used the same binning procedure as described in the main text. Under both the Pearson's correlation and Spearman's rank correlation metrics, the resulting correlation estimates are statistically significantly different from 0 for all but two animals (animal 2 and animal 4).

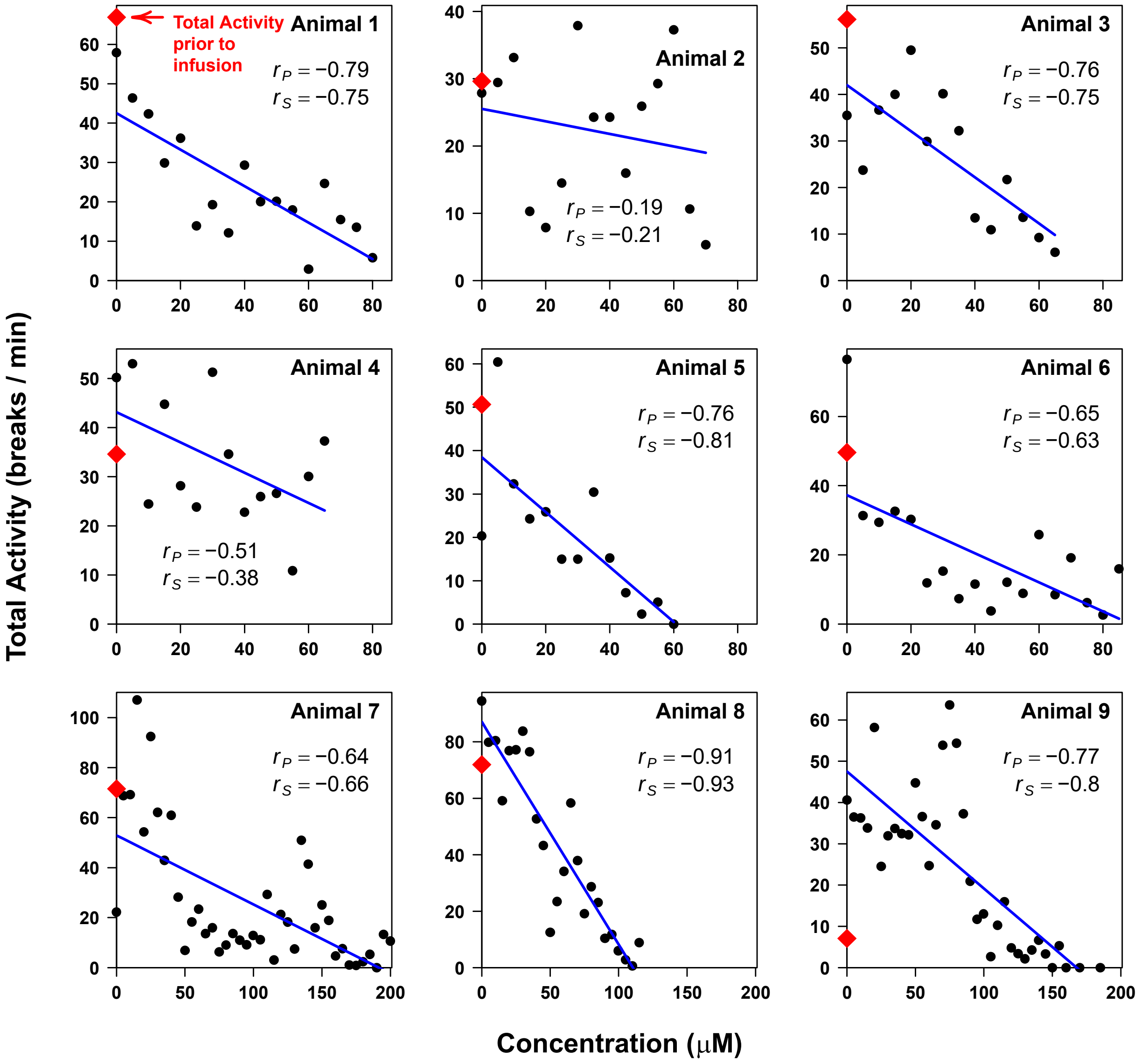

Figure SI8. Individual Subject Correlations between Neuropharmacokinetics and Total Activity during Procaine Exposure. Shown is total locomotor activity, which also decreases with increasing intracranial concentrations of the anesthetic procaine. The resulting correlation estimates are statistically significantly different from 0 under both metrics for all but two animals (animal 2 and animal 4).

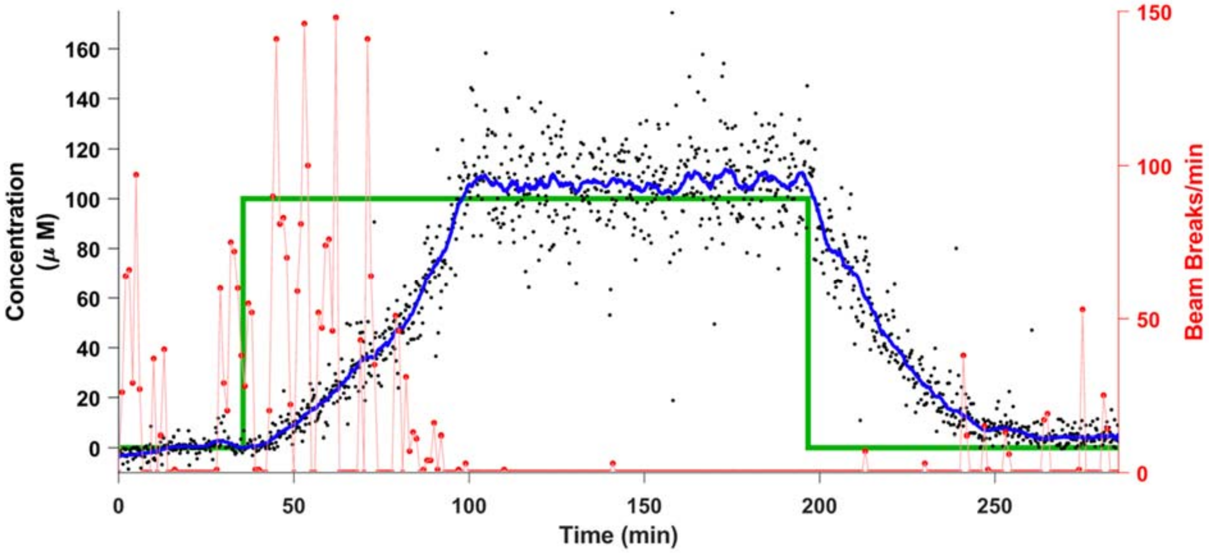

Figure SI9. Unfiltered Data for Closed-Loop Control Over In-Brain Procaine Control. Shown here is the raw data from our feedback control experiment (Fig. 5), prior to Hampel filtering to remove noise artifacts.

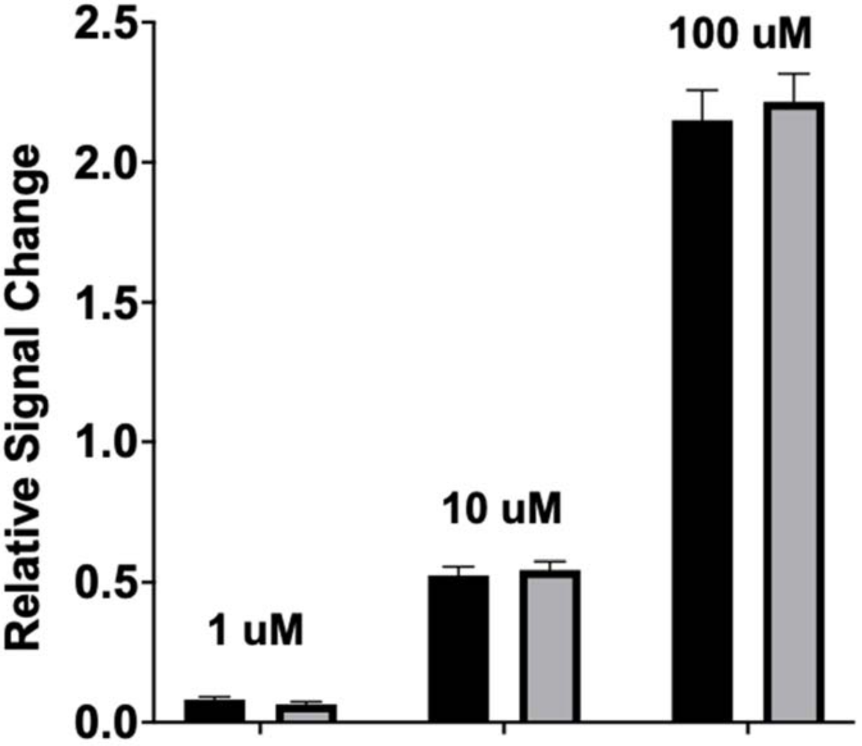

Figure SI10. Comparison of Target-Induced Relative Signal Change on Two- verses Three-Electrode EAB Configurations. Signal response of the EAB sensors does not vary between a two electrode (black) vs a three electrode (grey) system. Working electrodes (n=8) were alternatingly interrogated, in 1X PBS at room temperature, using either a stainless pseudo counter/reference (two electrode), or an Ag/AgCl reference and Pt counter electrode (three electrode) at various concentrations of procaine.

**Table SI1. The Neuropharmacokinetics and Modeling of Rearing during Procaine Exposure in Individual Subjects**						
Animal number	**Pharmacokinetics**			**Pharmacodynamics**		
	Time to first detection** (min)	C_{max} (µM)	$t_{1/2}$ (min)	Mean locomotion prior to infusion (breaks/min)	r_p	r_s
80 mg/kg dosing						
**1**	1.18	66.4 ± 0.3	13.8 ± 0.1	71.8	-0.76*	-0.71*
**2**	1.98	57.9 ± 0.3	11.2 ± 0.1	31.6	-0.37	-0.12
**3**	2.72	66.3 ± 0.3	14.3 ± 0.1	38.8	-0.69*	-0.73*
**4**	2.92	61.2 ± 0.3	15.3 ± 0.1	46.6	-0.29	-0.37
**5**	1.23	65.7 ± 0.3	10.9 ± 0.1	48.2	-0.87*	-0.86*
**6**	1.60	79.5 ± 0.3	8.6 ± 0.1	66.0	-0.72*	-0.58*
160 mg/kg dosing						
**7**	2.90	197.2 ± 0.3	15.9 ± 0.1	68.4	-0.53*	-0.60*
**8**	3.67	122.2 ± 0.2	19.6 ± 0.1	66.6	-0.93*	-0.91*
**9**	2.17	137.4 ± 0.2	17.7 ± 0.1	12.1	-0.70*	-0.68*

r_P denotes the estimate of the true Pearson's correlation coefficient ρ_P
r_S denotes the estimate of the true Spearman's correlation coefficient ρ_S
*Denotes estimated correlations are significantly different from 0 at a significance level α = 0.05
**Defined as the time taken to surpass the limit of detection, here defined as 3.7 µM (see main text)

**Table SI2. The Neuropharmacokinetics and Modeling of Total Activity during Procaine Exposure in Individual Subjects**						
Animal number	**Pharmacokinetics**			**Pharmacodynamics**		
	Time to first detection** (min)	C_{max} (μM)	$t_{1/2}$ (min)	Mean locomotion prior to infusion (breaks/min)	r_p	r_s
80 mg/kg dosing						
**1**	1.18	66.4 ± 0.3	13.8 ± 0.1	66.9	-0.79*	-0.75*
**2**	1.98	57.9 ± 0.3	11.2 ± 0.1	29.6	-0.19	-0.21
**3**	2.72	66.3 ± 0.3	14.3 ± 0.1	56.1	-0.76*	-0.75*
**4**	2.92	61.2 ± 0.3	15.3 ± 0.1	34.6	-0.51	-0.38
**5**	1.23	65.7 ± 0.3	10.9 ± 0.1	50.6	-0.76*	-0.81*
**6**	1.60	79.5 ± 0.3	8.6 ± 0.1	49.6	-0.65*	-0.63*
160 mg/kg dosing						
**7**	2.90	197.2 ± 0.3	15.9 ± 0.1	71.5	-0.64*	-0.66*
**8**	3.67	122.2 ± 0.2	19.6 ± 0.1	71.9	-0.91*	-0.93*
**9**	2.17	137.4 ± 0.2	17.7 ± 0.1	7.1	-0.77*	-0.80*

r_p denotes the estimate of the true Pearson's correlation coefficient ρ_P
r_s denotes the estimate of the true Spearman's correlation coefficient ρ_S
*Denotes estimated correlations are significantly different from 0 at a significance level α = 0.05
**Defined as the time taken to surpass the limit of detection, here defined as 3.7 μM (see main text)
